# PLA- and PLA/PLGA-Emulsion Composite Biomaterial Sheets for the Controllable Sustained Release of Hydrophilic Compounds

**DOI:** 10.3390/ma11122588

**Published:** 2018-12-19

**Authors:** Hitomi Moroishi, Seiichi Sonotaki, Yoshihiko Murakami

**Affiliations:** Department of Organic and Polymer Materials Chemistry, Tokyo University of Agriculture and Technology, 2-24-16 Naka-cho, Koganei-shi, Tokyo 184-8588, Japan; muralab-tuat@y3.dion.ne.jp (H.M.); s162591s@st.go.tuat.ac.jp (S.S.)

**Keywords:** sheet, emulsion, sustained release, biomaterial, PLA, PLGA

## Abstract

In the present study, by spin-coating a solution containing w/o (water-in-oil) emulsions and hydrophobic polymers, we obtained sheets possessing uniformly dispersed w/o emulsions. We performed release experiments for more than 100 days and clarified the effects of the number of layers, the sheet-forming polymers (polylactide (PLA), poly(lactic-*co*-glycolic acid (PLGA)), the ratio of organic solvent to water, and the composition of block copolymers on the release properties of the sheets. For a variety of sheets, we successfully achieved the sustained release of compounds from the sheets for 100–150 days. The sustained-release of compounds occurred because the compounds had to diffuse into polymer networks after their release from the emulsions. Interestingly, we observed an inflection point in the release profiles at around 50 days; that is, the sheet exhibited a “two-step” release behavior. The results obtained in the present study provide strong evidence for the future possibility of the time-programmed release of multiple compounds from sheets.

## 1. Introduction

Developing a host of biomaterials that stably incorporate hydrophilic biomacromolecules (e.g., proteins) and release them in a sustained manner is important for the effective differentiation and growth of cells in tissue engineering. In general, it is difficult to control the sustained-release property of gel-type biomaterials because the release of compounds from hydrogels depends mainly on two phenomena: the degradation of the hydrogels and the diffusion of the compounds through the hydrogels. Both these phenomena can be partially regulated by tuning the hydrogels’ properties, such as the charge and crosslink density. However, it is difficult to control the diffusion and the degradation precisely, because the factors affecting them, such as pH and body-fluid levels, are variable in the body. By contrast, sheets have several advantages over conventional gels: (1) sheets have a large contact area relative to drug-targeting sites, (2) the shape and size of sheets are easily adjustable, (3) sheets perform well in surgery, and (4) molecular interactions, such as the Van der Waals force, can facilitate the gentle adhesion of sheets to body tissues without an inflammatory reaction at the tissue surface. The properties of sheets, including their flexibility, strength, biocompatibility, and degradation rate, can be altered through both the blending of several substances [1,2,3,4,5] and the formation of porosities [1,6,7]. Because of these properties, sheets are attractive for use in biomedical applications, such as wound-dressing [8,9,10,11,12] and sustained drug release [13,14,15,16,17,18]. Although there have been a variety of reports on hydrophobic sheets stating that they can incorporate and release hydrophobic drugs, the incorporation and release of hydrophilic compounds from sheets remains difficult [19,20]. Therefore, there is an urgent need for the development of a novel biomaterial that can incorporate and release hydrophilic compounds.

**Figure 1:**
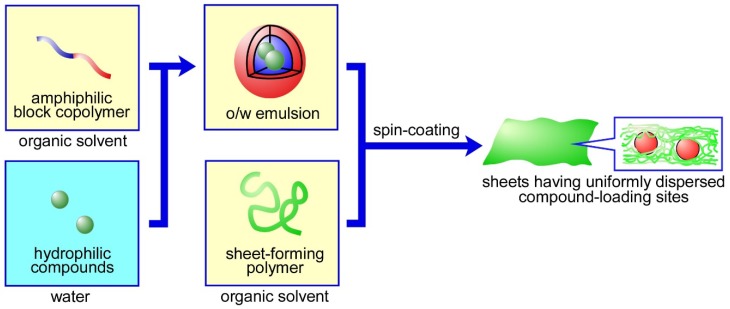
The preparation of sheets with uniformly dispersed compound-loading sites.

To achieve the successful incorporation and release of drugs, we have developed a novel approach for the construction of functional biomaterials. We have applied this approach to the chemical or physical conjugation of functional units (such as amphiphilic block copolymers or their self-assemblies) to base materials, including gels [21,22,23,24,25,26,27,28], sheets [29,30,31], and particles [32,33,34,35,36]. For example, the covalent incorporation of trilayered polymeric micelles [37,38] with hydrophilic inner cores into gels gave them the ability to release hydrophilic compounds [24]. Of the three types of hybrid materials, sheets can—using our approach—be easily prepared to contain compound-loading sites within them. Basically, amphiphilic block copolymers are assembled and consequently result in stable droplets (w/o emulsion) with a hydrophilic inner core in organic solvents. By spin-coating a solution containing a w/o (water-in-oil) emulsion and hydrophobic polymers, we can easily obtain sheets possessing uniformly dispersed w/o emulsions (Figure 1). We have reported the effects of preparation conditions on, for example, the stability [29,30] and the dispersity [30] of inner cores formed from w/o emulsions, the rheological properties [31], and the degradation properties [31]. In addition, although we conducted preliminary release experiments [29,31], the release properties of the sheets were not fully determined. In the present study, we performed release experiments for more than 100 days and clarified, in detail, the effects that the number of layers, the sheet-forming polymers, the ratio of organic solvent to water, and the composition of block copolymers on the release properties of sheets. Interestingly, we found that the sheets successfully exhibit a “two-step” release behavior. To our knowledge, this is the first report on sheets that exhibit a two-step release behavior from uniformly dispersed compound-loading sites.

## 2. Materials and Methods

### 2.1. Materials

Amphiphilic block copolymers and methoxy-terminated poly(ethylene glycol)-*block*-poly(ε-caprolactone) (PEG-*b*-PCL), with different compositions, were previously synthesized by anionic ring-opening polymerization of both ethylene oxide and ε-caprolactone [29], as listed in Table 1. Fluorescein isothiocyanate-dextran (FITC-dex, average molecular weight: 20,000) was purchased from Sigma-Aldrich. Poly(vinyl alcohol) (PVA, degree of polymerization: 500, saponification degree: 86–90 mol%) and PLGA (poly(lactic-*co*-glycolic acid), monomer ratio of lactide to glycolide: 3, average molecular weight: 20,000) were purchased from Wako Pure Chemical Industries, Ltd. (Osaka, Japan). PLA (average molecular weight: 300,000) was purchased from Polysciences Inc. (Warrington, PA). All other reagents were of analytical grade and were used without further purification.

### 2.2. Preparation of the Sheets

We prepared sheets in which either w/o emulsions or w/o emulsions containing fluorescent hydrophilic polymers were dispersed. FITC-dextran was used as a fluorescent indicator for observing the release properties of the sheets. The organic solvent used was a dichloromethane-toluene mixed organic solvent, whose density was adjusted to 1.00 g/cm^3^. First, in the presence of the polymeric surfactant PEG-*b*-PLA (10 w/v%), the w/o emulsion (1 mL) was prepared by sonicating both the organic solvent and an aqueous PVA solution (1 w/v%) for 5 min in an ice bath (PVA was used as an emulsion stabilizer). The volume ratio of the organic solvent over the aqueous solution was 399 or 39. Next, the obtained w/o emulsion (1 mL) was added to 10 mL of the organic solvent containing sheet-forming polymers (PLA (1–3 w/v%) or PLA (2 w/v%) with PLGA (1–4 w/v%)). The concentration of each polymer corresponded to the value in the final solution after mixing the emulsion and organic solvent. We then obtained a thin sheet by spin-coating 1 mL of the solution at 2000 rpm for 5 min on an aluminum substrate, which was cleaned with MilliQ water and acetone prior to use (if desired, multi-layered sheets can be prepared through a repetition of the procedures).

### 2.3. Characterization of the Emulsions and Sheets

The stability of the emulsion was determined through static observation and dynamic light scattering (DLS), using the Zetasizer Nano-ZS (Malvern Instruments, Worcestershire, UK). The thickness of the sheet was determined by a laser confocal displacement meter (LT-9000, KEYENCE Co., Tokyo, Japan). The dispersion of the emulsion in the sheets was observed using a fluorescent microscope (BZ-9000, KEYENCE Co., Tokyo, Japan, λ_ex_ = 470 nm, λ_em_ = 535 nm).

### 2.4. Release of FITC-Dex from the Sheets in Which W/O Emulsions Containing FITC-Dex Were Dispersed

The sheets (for which the layer number was 11) obtained in the previous section were immersed in PBS buffer (100 mL, 10 mM, pH 7.4, 37 °C). The release of FITC-dex from the sheets was monitored using a fluorescence spectrometer (λ_ex_ = 495 nm, λ_em_ = 519 nm).

## 3. Results and Discussion

### 3.1. Preparation and Characterization of the Sheets in Which W/O Emulsions Were Dispersed

In the present study, we aimed to develop biomaterial sheets with uniformly dispersed inner spaces, in which it would be possible to load hydrophilic compounds by spin-coating a solution containing both w/o emulsions prepared from assembled block copolymers (PEG-*b*-PLA) and sheet-forming hydrophobic polymers (PLA and PLGA). For the polymeric units that form the block copolymers, we selected PEG and PCL, because PEG is a biocompatible polymer that has been used as a non-immunogenic modifier for proteins and drugs [39,40,41,42,43], whereas PCL is a biodegradable polymer that has been used in the preparation of various biomaterials [44,45,46,47]. We used PLA and PLGA as sheet-forming polymers because PLA [48,49,50] and PLGA [51,52,53] have been frequently used for the preparation of polymeric biomaterials, because of their biocompatibility and easily controllable degradation properties. The w/o emulsions had to be highly stable in order to form uniformly dispersed inner spaces in the sheets, during the sheet preparation process. An emulsion is a system consisting of two immiscible liquid phases, one of which (the dispersed phase) is dispersed throughout the other (the continuous phase) in stable, small droplets. All the droplets will clump together and phase separation will occur over time because the emulsion is usually unstable. Macroscopic separation of the phases is commonly prevented by using suitable organic solvents, stabilizers, surfactants, and so on. One of the easiest methods to increase the stability of an emulsion is the use of mixed organic solvents, whose density is adjusted to 1.00 g/cm^3^, as the phase separation of the emulsion is unlikely to occur when the difference in density between the two the phases is nearly equal to zero, because of the thermodynamically high stability of the emulsion [29,32]. Through static observations of the stability of the w/o emulsions, we found that an unstable emulsion was obtained when the block copolymers coded as 1.6k–0.7k and 3.0k–0.6k were used. By contrast, a stable emulsion was formed when the block copolymers coded as 1.5k–1.9k, 3.2k–1.9k, 2.9k–3.8k, and 4.2k–8.1k were used (here, we defined an “unstable” emulsion as one that gradually aggregates and causes a phase separation over time, whereas a “stable” emulsion is one that maintains its diameter and does not induce a phase separation as time passes). Using DLS to identify the size distribution of the emulsions, we found that the average diameters of the emulsions formed from the block copolymers coded as 1.5k–1.9k, 3.2k–1.9k, 2.9k–3.8k, and 4.2k–8.1k were 450, 320, 870, and 520 nm, respectively. The results of the static observations suggested that the stability of the w/o emulsions decreased as the *M*_n_ of PCL decreased, presumably because fewer PCL chains weaken both the formation of emulsion droplets and repulsion between droplets. We used the block copolymers coded as 3.2k–1.9k in the following experiments unless otherwise noted.

**Figure 2:**
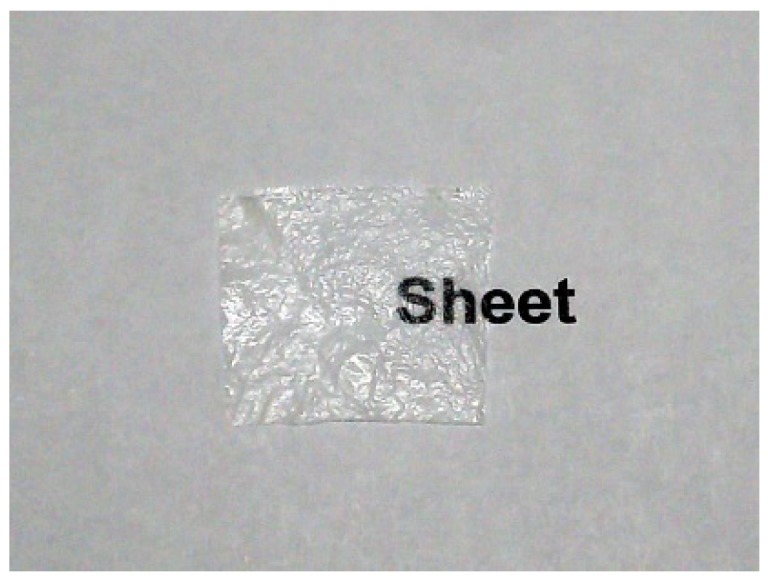
The appearance of a sheet with uniformly dispersed compound-loading sites (the letters “S” and “h” in the word “Sheet”, written on paper, can be seen through the transparent sheet).

**Figure 3:**
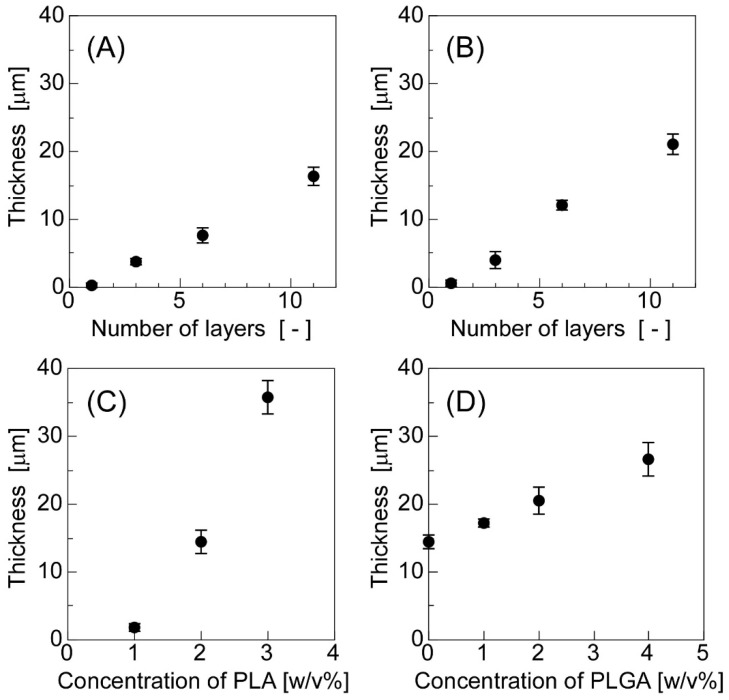
The effects of the number of layers and polymer concentrations on the thickness of the sheets: (**A**) sheets prepared with a PLA (2 w/v%) solution, (**B**) sheets prepared with a PLA (2 w/v%) + PLGA (2 w/v%) solution, (**C**) sheets prepared with a PLA solution (number of layers: 11), and (**D**) sheets prepared with a PLA (2 w/v%) + PLGA solution (number of layers: 11). The polymer concentrations indicate the values at the preparation stage of the emulsions.

Transparent, thin, flexible, free-standing sheets were successfully obtained by spin-coating solutions containing both emulsions and sheet-forming polymers (Figure 2). Figure 3 shows the effects of the number of layers and polymer concentrations used in the sheet preparation stage on the sheet thickness. The thickness increased as the number of layers increased, when the sheets were prepared from PLA or PLA with PLGA (Figure 3A,B). The thickness also increased as the polymer concentrations increased (Figure 3C,D). In both (C) and (D), the number of layers was 11. As shown in Figure 3C, the thickness of the sheets drastically increased as the concentration of PLA increased, because the solution used (the concentration of PLA was over 2 w/v%) was viscous and difficult to spread during the spin-coating process. By contrast, the solution of PLGA—whose average molecular weight was 20,000—was not as viscous; thus, the combination of PLGA and PLA had smaller effects on the sheet thickness than the PLA alone. Furthermore, the addition of PLGA made the sheets more flexible because PLA is crystalline, whereas PLGA is amorphous [54,55]. In two cases, when PLA alone (Figure 3A) and when both PLA and PLGA served as sheet-forming polymers (Figure 3B), we obtained flexible, thin, single-layered sheets with a diameter less than 1 µm.

### 3.2. The Release of FITC-Dex from the Sheets in Which W/O Emulsions Containing FITC-Dex Were Dispersed

To determine the factors that affect the release properties of the sheets, we prepared sheets in which w/o emulsions containing a hydrophilic polymer, FITC-dextran, were dispersed, and we evaluated the release of FITC-dextran from the sheets. The release of FITC-dex from the sheets was monitored using a fluorescence spectrometer for 100–150 days.

**Figure 4:**
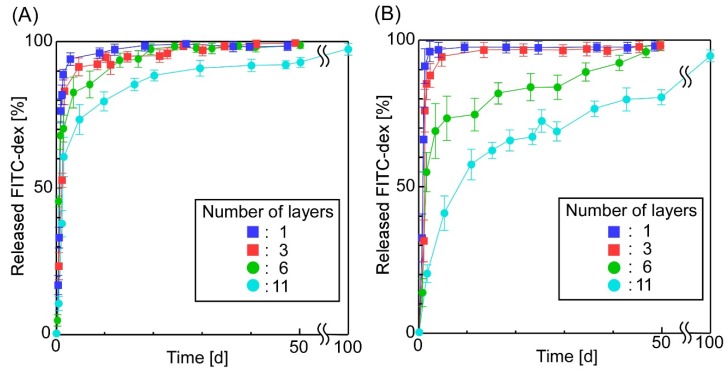
The effect of the number of layers on the release properties of the sheets (the sheet-forming polymers were (**A**) PLA (2 w/v%) and (**B**) PLA (2 w/v%) + PLGA (2 w/v%); number of layers: 11).

**Figure 5:**
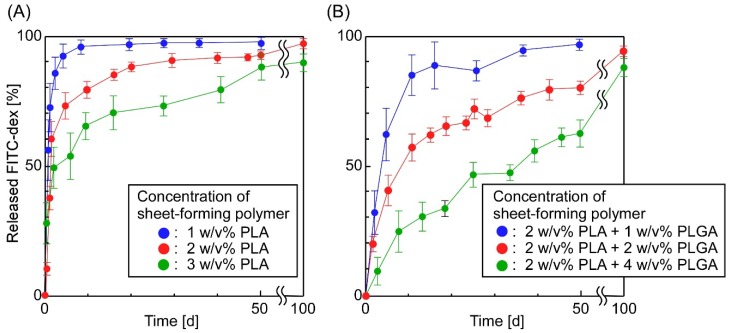
The effect of the concentration of sheet-forming polymers on the release properties of the sheets (the sheet-forming polymers were (**A**) PLA and (**B**) PLA (2 w/v%) + PLGA; number of layers: 11).

Figure 4 shows the effect of the number of layers on the sheets’ release properties. We found that the number of layers forming the sheets affected the release behavior of FITC-dex from the sheets: an increase in layers could suppress the initial burst release of FITC-dex. Almost the same phenomena were observed when determining the effects of the concentrations of sheet-forming polymers on the release properties of the sheets (Figure 5): An increase in the concentration of sheet-forming polymers could suppress the initial burst release, and decrease the total release of FITC-dex. Presumably, these results can be explained by three phenomena: (1) an increase in the number of layers suppressed the access of the incorporated compounds to the bulk solution because the sheets’ thickness increased, (2) an increase in sheet-forming polymers suppressed the diffusion of the incorporated compounds in the sheets, and (3) an increase in sheet-forming polymers suppressed the mobility of the emulsions’ surface, making it more difficult to release compounds from the emulsions. These results suggested that the release behavior of compounds from the sheets could be controlled by tuning both the concentration and the composition of sheet-forming polymers. Similar results have been reported concerning drug-incorporated sheets: The concentration and drying method of sheet-forming polymers (collagen) affected the release of proteins such as human growth hormone (hGH) [56] and interferon [57], the composition of multi-block poly(ether-ester) matrices affected the release of proteins [58], and thermally induced cross-linking of sheets suppressed the release of hydrophobic drugs [59].

**Figure 6:**
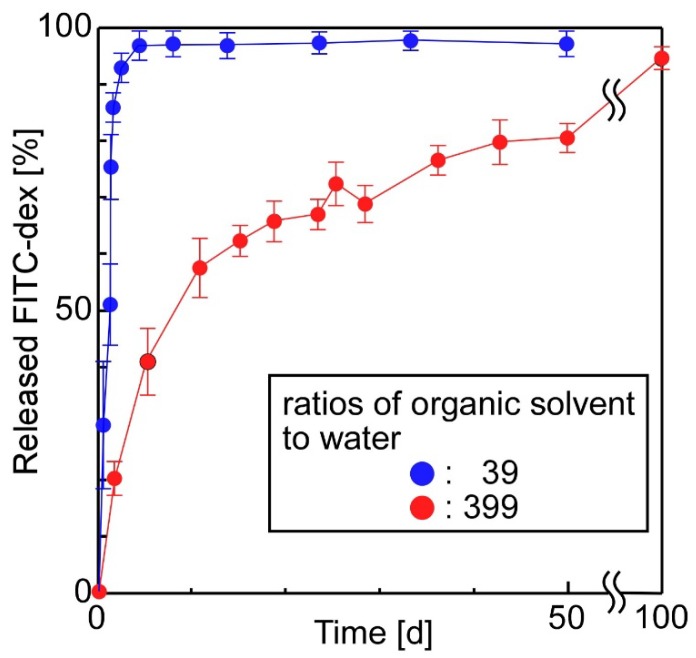
The effect of the ratio of organic solvent to water in the preparation stage of emulsions on the release properties of the sheets (the sheet-forming polymers were PLA (2 w/v%) + PLGA (2 w/v%); number of layers: 11).

Figure 6 shows the effect of the ratios of organic solvent to water in the preparation stage of emulsions on the release properties of the sheets. As shown, the sheets prepared from an emulsion with a ratio of organic solvent to water of 39, exhibited faster release than sheets prepared from an emulsion with a ratio of 399. In fact, in the former case, almost all the FITC-dex was released from the sheet within a few days because the stability of the w/o emulsions decreased as the volume fraction of water increased. As the volume fraction of water increased, the diameter of water droplets increased, and consequently, the density of emulsions’ shells (PCL) decreased. These phenomena induced the fast release of compounds from the emulsions.

**Figure 7:**
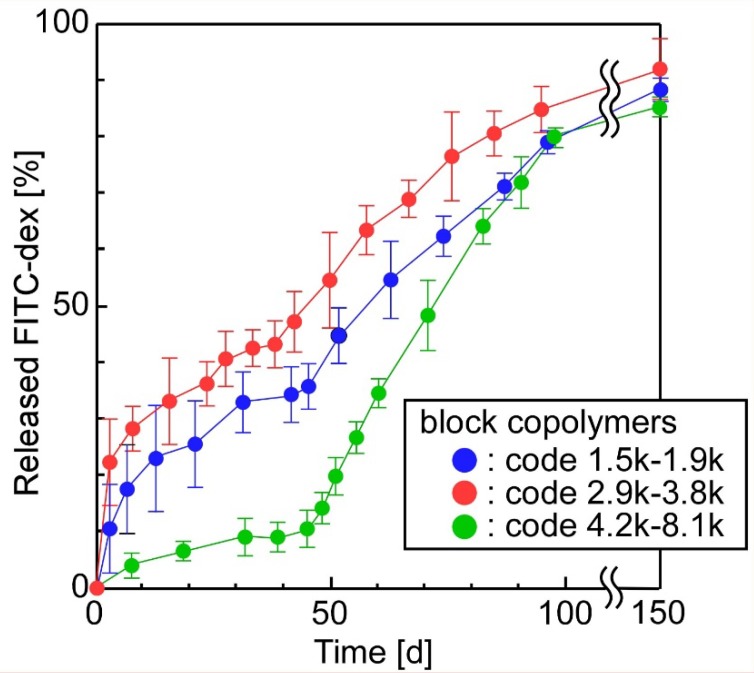
The effect of the composition of block copolymers on the release properties of the sheets (the sheet-forming polymers were PLA (2 w/v%) + PLGA (2 w/v%); number of layers: 11).

Finally, we evaluated the effect of the composition of block copolymers on the release properties of sheets prepared, using PLA and PLGA as sheet-forming polymers. Here, we used block copolymers, where the *M*_n_ of PEG was lower than the *M*_n_ of PCL (code 1.5k–1.9k, code 2.9k–3.8k and code 4.2k–8.1k). Figure 7 shows that the release of FITC-dex was suppressed when the block copolymers coded as 1.5k–1.9k, 2.9k–3.8k and 4.2k–8.1k were used to form emulsions, but was not suppressed when the block copolymer coded as 3.2k–1.9k was used (Figure 5A). This difference in the release behavior was due to the composition of block copolymers; that is, the block copolymers (coded as 1.5k–1.9k, 2.9k–3.8k and 4.2k–8.1k) with a higher *M*_n_ of PCL than that of PEG presumably formed more stable emulsions than the block copolymers (coded as 3.2k–1.9k) with a lower *M*_n_ of PCL than that of PEG. Furthermore, the compound-release rates of the sheets ranked in descending order, according to the block copolymers’ code, as 2.9k–3.8k, 1.5k–1.9k, and 4.2k–8.1k. These results support the assumption that PCL, with its higher molecular weight, formed emulsions with outer high-density PCL layers, and consequently, the increased outer PCL layers suppressed the release of FITC-dex from the emulsions. In another report [60], poly(lactide-*co*-glycolide)-grafted dextran microspheres exhibited slow-release properties when hydrophobic units of grafted polymers increased. These results (including our results) suggest that controlling the amounts of hydrophobic and hydrophilic units at optimal levels helps to regulate the release properties of the materials.

Among the three types of sheet tested in Figure 7, the one whose emulsions were prepared from the block copolymer coded as 4.2k–8.1k, released FITC-dex slowly for 50 days and, consequently, released FITC-dex quickly at zero-order for 100 days. Interestingly, we observed an inflection point in the release profiles at around 50 days; that is, the sheet exhibited a “two-step” release behavior. We hypothesize that this interesting phenomenon occurred because the two following steps were necessary in order to release the incorporated compounds from the sheets: (1) in the first period (0–50 days), the compounds had to release from the emulsion across the outer high-density PCL shells and (2) in the next period (50–100 days), the compounds had to diffuse into the polymer networks after the compounds’ release from emulsions that were uniformly dispersed in the sheets. A similar slight “two-step” release behavior was observed with the block copolymers coded as 1.5k–1.9k and 2.9k–3.8k at 40 h. These results suggest that the “two-step” release behavior can clearly be observed when block copolymers having a higher *M*_n_ of PCL are used, because the increase in the thickness of the PCL outer shell suppresses the initial release of FITC-dex from the inner space of the emulsions.

Combination therapies, in which multiple drugs with different therapeutic effects are used together, have garnered attention as a method for enhancing therapeutic performance in clinical treatments [61,62]. A variety of multiple drug release systems have been proposed, including a silk fibroin protein scaffold in which calcium alginate is embedded [63], electrospun nanofibers [64], a physically formed polymeric micelle-hydrogel composite [65], and a covalently formed polymeric micelle-hydrogel composite [26]. For example, multilayered drug-loaded poly(L-lactide-*co*-ε-caprolactone) nanofiber meshes were prepared using sequential electrospinning. The mesh consisted of four layers: a drug-loaded mesh, a barrier mesh, another drug-loaded mesh, and a basement mesh [64]. The release profiles of the second drug were regulated by the optimal design of the barrier-mesh. There is significant demand for the continual development of biomaterials designed for the dual release of compounds. Unlike previously reported materials for the dual (or multiple) release of compounds, our sheets (as shown in Figure 7) simultaneously offer three distinct advantages: (1) the preparation requires only physical processes (no chemical reaction is necessary), (2) hydrophilic compounds can be encapsulated despite the hydrophobic nature of the sheets, (3) the time-programmed release of multiple compounds is possible because the release profiles include two steps.

## 4. Conclusions

In the present study, by spin-coating a solution containing w/o emulsion and hydrophobic polymers, we obtained sheets with uniformly dispersed w/o emulsions. We performed release experiments for more than 100 days and clarified the effect that four variables—the number of layers, the type of sheet-forming polymer, the ratio of organic solvent to water, and the composition of block copolymers—had on the release properties of PLA- or PLA/PLGA-emulsion composite sheets. For a variety of sheets, we successfully achieved the sustained-release of compounds from the sheets for 100–150 days. The sustained release of the compounds was successfully achieved because entangled sheet-forming polymer networks suppressed the diffusion of the compounds. Interestingly, we observed an inflection point in the release profiles at 50 days; that is, the sheet exhibited a “two-step” release behavior. Although further studies on biocompatibility, cytotoxicity, and cell-material interactions are necessary before biomedical use, the results obtained in the present study strongly suggest that the time-programmed release of multiple compounds from sheets will be a reality in the near future.

## Figures and Tables

**Table 1:** The different compositions of poly(ethylene glycol)-*block*-poly(ε-caprolactone) (PEG-*b*-PCL) used in this study.

Code ^1^	PEG (Including Methoxy Terminus)		PCL	PEG-*b*-PCL	
	*M*_n_^2^	*M*_w_/*M*_n_^2^	*M*_n_^3^	*M*_n_^2,3^	*M*_w_/*M*_n_^2^
1.6k–0.7k	1600	1.11	700	2300	1.11
1.5k–1.9k	1500	1.10	1900	3400	1.24
3.0k–0.6k	3000	1.05	600	3600	1.08
3.2k–1.9k	3200	1.04	1900	5100	1.11
2.9k–3.8k	2900	1.05	3800	6700	1.13
4.2k–8.1k	4200	1.04	8100	12,300	1.19

^1^ Code *a*-*b* represents a block copolymer with a composition of PEG *M*_n_ (*a*) and PCL *M*_n_ (*b*), ^2^ GPC, ^3 1^H NMR.
